# Diffuse alveolar damage associated mortality in selected acute respiratory distress syndrome patients with open lung biopsy

**DOI:** 10.1186/s13054-015-0949-y

**Published:** 2015-12-01

**Authors:** Kuo-Chin Kao, Han-Chung Hu, Chih-Hao Chang, Chen-Yiu Hung, Li-Chung Chiu, Shih-Hong Li, Shih-Wei Lin, Li-Pang Chuang, Chih-Wei Wang, Li-Fu Li, Ning-Hung Chen, Cheng-Ta Yang, Chung-Chi Huang, Ying-Huang Tsai

**Affiliations:** 1 Department of Pulmonary and Critical Care Medicine, Chang Gung Memorial Hospital, Chang Gung University College of Medicine, Linkou, No. 5, Fu-Shing St, Kwei-Shan, Taoyuan, 886 Taiwan; 2 Department of Respiratory Therapy, Chang Gung Memorial Hospital, Chang Gung University College of Medicine, Taoyuan, Taiwan; 3 Department of Respiratory Therapy, Chang-Gung University College of Medicine, Taoyuan, Taiwan; 4 Department of Pathology, Chang Gung Memorial Hospital, Chang Gung University College of Medicine, Taoyuan, Taiwan; 5 Department of Pulmonary and Critical Care Medicine, Chang Gung Memorial Hospital, Chang Gung University College of Medicine, Chiayi, Taiwan

**Keywords:** Continuous Positive Airway Pressure, Acute Respiratory Distress Syndrome, Sequential Organ Failure Assessment Score, Acute Respiratory Distress Syndrome Patient, Diffuse Alveolar Damage

## Abstract

**Introduction:**

Diffuse alveolar damage (DAD) is the pathological hallmark of acute respiratory distress syndrome (ARDS), however, the presence of DAD in the clinical criteria of ARDS patients by Berlin definition is little known. This study is designed to investigate the role of DAD in ARDS patients who underwent open lung biopsy.

**Methods:**

We retrospectively reviewed all ARDS patients who met the Berlin definition and underwent open lung biopsy from January 1999 to January 2014 in a referred medical center. DAD is characterized by hyaline membrane formation, lung edema, inflammation, hemorrhage and alveolar epithelial cell injury. Clinical data including baseline characteristics, severity of ARDS, clinical and pathological diagnoses, and survival outcomes were analyzed.

**Results:**

A total of 1838 patients with ARDS were identified and open lung biopsies were performed on 101 patients (5.5 %) during the study period. Of these 101 patients, the severity of ARDS on diagnosis was mild of 16.8 %, moderate of 56.5 % and severe of 26.7 %. The hospital mortality rate was not significant difference between the three groups (64.7 % vs 61.4 % vs 55.6 %, p = 0.81). Of the 101 clinical ARDS patients with open lung biopsies, 56.4 % (57/101) patients had DAD according to biopsy results. The proportion of DAD were 76.5 % (13/17) in mild, 56.1 % (32/57) in moderate and 44.4 % (12/27) in severe ARDS and there is no significant difference between the three groups (p = 0.113). Pathological findings of DAD patients had a higher hospital mortality rate than non-DAD patients (71.9 % vs 45.5 %, p = 0.007). Pathological findings of DAD (odds ratio: 3.554, 95 % CI, 1.385–9.12; p = 0.008) and Sequential Organ Failure Assessment score on the biopsy day (odds ratio: 1.424, 95 % CI, 1.187–1.707; p<0.001) were significantly and independently associated with hospital mortality. The baseline demographics and clinical characteristics were not significantly different between DAD and non-DAD patients.

**Conclusions:**

The correlation of pathological findings of DAD and ARDS diagnosed by Berlin definition is modest. A pathological finding of DAD in ARDS patients is associated with hospital mortality and there are no clinical characteristics that could identify DAD patients before open lung biopsy.

## Introduction

In 1994 the American-European Consensus Conference (AECC) released its definitions of acute lung injury (ALI) and acute respiratory distress syndrome (ARDS) [1]. Several studies had challenged the AECC criteria for the diagnosis of ARDS because of its many limitations [2–7]. To provide a more reliable definition of ARDS, an expert panel revisited the AECC definition and released the Berlin definition of ARDS [8]. However, the pathological diagnosis of ARDS using lung biopsy was not included in the Berlin definition because of the controversial definition of the pathology and concern about surgical complications [9].

It is essential to clarify the diagnosis of ARDS and to initiate effective treatments such as low tidal volume and prone positioning to improve clinical outcome [10–12]. The current clinical definition of ARDS reflects only nonspecific functional or physiological abnormalities rather than pathological abnormality. Since ARDS may occur with other pathologic findings, it is uncertain if the same management should be applied to all patients. The heterogeneity of patients with ARDS included in therapeutic trials remains a challenge when interpreting results from such trials [13]. Therefore, open lung biopsy sometimes has been performed to better define the pathology and to guide therapeutic management of selected patients with ARDS [14–17].

ARDS is a form of ALI characterized by severe inflammation with increasing epithelial and endothelial permeability [18]. The pathologic hallmark of ARDS is diffuse alveolar damage (DAD) characterized by hyaline membrane formation, lung edema, inflammation, hemorrhage and alveolar epithelial cell injury [19, 20]. A post mortem study showed that 66 % of 127 patients who met the AECC definition of ARDS had typical DAD findings [21]. Another study reported that of the 64 patients diagnosed as having ARDS using AECC criteria, 50 % (32/64) had DAD findings at autopsy [22]. A large retrospective study in Spain revealed that among 356 patients who met the Berlin definition of ARDS, 45 % had DAD at autopsy [23, 24]. However, autopsy results are based on results of analysis of samples from deceased patients and thus, there might be differences to results of analysis in the living. The purpose of this study was to investigate the role of DAD in patients with ARDS defined by the Berlin definition on open lung biopsy.

## Methods

### Patient population

Our study was approved by the Institutional Review Board of Chang Gung Memorial Hospital which waived the need for informed consent due to the retrospective nature of the study. Chang Gung Memorial Hospital is a tertiary care referral center with a 3700-bed general ward, and a 278-bed adult ICU. The hospital charts of all patients with ARDS who underwent open lung biopsy from January 1999 to January 2014 were reviewed. All patients who were given a discharge diagnosis code of 518.82 (according to the *International Classification of Diseases, Ninth Revision, Clinical Modification*) were reviewed for possible inclusion in this study.

Using the Berlin definition, ARDS was characterized as mild if the arterial partial pressure of oxygen/inspired oxygen fraction (PaO_2_/FiO_2_) was between 201 and 300 mm Hg, moderate if PaO_2_/FiO_2_ was between 101 and 200 mm Hg, and severe if PaO_2_/FiO_2_ was less than or equal to 100 mm Hg, in all cases using either continuous positive airway pressure (CPAP) or positive end-expiratory pressure (PEEP) of at least 5 cm H_2_O [8]. A total of 1,838 patients with ARDS were identified by the chart sheets and open lung biopsies were performed on 101 patients (5.5 %). Those patients with a PaO_2_/FiO_2_ <200 while on CPAP, or those treated with another noninvasive ventilation approach classified as as having moderate or severe ARDS were excluded. The ARDS severity was characterized at the time of diagnosis. These 101 patients included patients previously identified using the AECC definition of ARDS, but reanalyzed in this study according to the Berlin definition of ARDS [16, 25].

### Microbiological examinations before open lung biopsy

The results of cultures from blood, sputum, transtracheal aspiration, and pleural effusion were recorded. The location for bronchoalveolar lavage (BAL) sampling was decided on the basis of findings from high-resolution computed tomography (HRCT) of the chest or chest x-ray (CXR), if HRCT was not available. Each specimen was examined for bacteria including Legionella, Mycoplasma pneumoniae, Pneumocystis jiroveci, and Mycobacteria, and for fungi and viruses (including cytomegalovirus, influenza virus, parainfluenza virus, adenovirus, herpes simplex virus, respiratory syncytial virus, and coxsackie virus). Specimens were also sent for cytology and iron stain analysis. BAL results were deemed positive when at the minimum one microorganism had grown to a concentration greater than 10^4^ colony-forming units/mL. All specimen examinations were performed within 24 hours of open lung biopsy.

### Open lung biopsy

Open lung biopsy was indicated when ARDS was suspected to be noninfectious, there was no obvious risk factor, and if there was a possible indication for corticosteroid therapy based on clinical presentation with rapid progression, relative symmetric distribution of infiltrates on CXR, and predominant ground-glass attenuation on HRCT of the chest. Informed consent for surgical lung biopsy was obtained from each patient’s family before surgery.

Open lung biopsy was performed in an operating room or at the bedside in the ICU. The lobe of the lung biopsy was chosen based on the presence of a new or progressive lesion identified on HRCT of the chest or CXR. While under general anesthesia, open lung biopsy was performed using either video-assisted thoracoscopic surgery (VATS) or a 5-cm thoracotomy, depending on the patient’s tolerance. For VATS and thoracotomy, an endoscopic stapler-cutter was used to secure the pulmonary margins. To avoid the risk of transfer to the operating room, the timing of bedside open lung biopsy was considered when the given FiO_2_ reached 1 with a PEEP above 12 cm H_2_O. Each tissue specimen was cultured and examined by pathologists.

### Evaluation of pathological appearances

Pathological criteria for the diagnosis of pneumonia involved severe neutrophil infiltration in the interstitium and intra-alveolar spaces, particularly around terminal bronchioles. The pathological criteria for the diagnosis of ALI and DAD included the presence of pulmonary inflammatory infiltrates and presence of hyaline membrane formation and at least one of the following: intra-alveolar edema, alveolar type I cell necrosis, alveolar type II cell proliferation progressively covering the denuded alveolar-capillary membrane, interstitial proliferation of fibroblasts and myofibroblasts, or organizing interstitial fibrosis [19, 20, 26].

Our strategy for mechanical ventilation of patients with ARDS consisted of an initial low tidal volume of 6 to 8 mL/kg of predicted body weight for either volume-controlled or pressure-controlled ventilation. Ventilatory adequacy was monitored by arterial blood gas measurements, with the ventilator settings changed as needed. The PEEP levels were set according to a lower PEEP and FiO_2_ strategy or at least 2 cm H_2_O above the lower inflection point derived from the P-V tool maneuvers of the ventilator [27]. The plateau airway pressure was maintained below 30 cm H_2_O combined the PEEP setting and low tidal volume strategy. Pulse oximeter was used to monitor oxygen saturation and the FiO_2_ was adjusted to maintain SpO_2_ above 90 %. The plateau airway pressure was tried to avoid raising above 30 cm H_2_O.

### Clinical data collection

The following data were collected from the hospital chart of each patient and analyzed: age, sex, underlying diseases, acute physiology and chronic health evaluation (APACHE) II score on the day of ICU admission [28], sequential organ failure assessment (SOFA) score on the day of ICU admission and the day of open lung biopsy [29], lung injury score (LIS) [30], PaO_2_/FiO_2_ ratio, PEEP, tidal volume, diagnostic procedures before open lung biopsy (HRCT or BAL), complications related to surgery (i.e., postoperative air leak, pneumothorax, subcutaneous emphysema, bleeding, and wound infection), pathological diagnosis, hospital mortality, and therapeutic alterations.

Postoperative therapeutic alterations indicated that the results of open lung biopsy had led to the addition of a new therapy, or the original therapy had been stopped. Immunocompromised patients were defined as follows: presence of HIV infection, recipient of solid organ transplantation, recipient of hematopoietic stem cell transplantation (HSCT), recipient of chemotherapy, and recipient of long-term systemic corticosteroids for more than 2 weeks.

### Statistical analysis

All statistical analyses were performed using the SPSS statistical package (SPSS for Windows, SPSS Inc., Chicago, IL, USA). All values are reported as means ± SD. Categorical data were tested using the chi-square test (or Fisher’s exact test when the expected number of events was fewer than five). Risk factors for hospital mortality were analyzed by univariate analysis, and the variables statistically significant (*p* <0.05) in the univariate analysis were included in the multivariate analysis by applying multiple logistic regression based on backward elimination of data. The Hosmer-Lemeshow goodness-of-fit test was used for calibration when evaluating the number of observed and predicted deaths in risk groups for the entire range of death probabilities. A *p* value <0.05 was considered statistically significant.

## Results

**Fig. 1:**
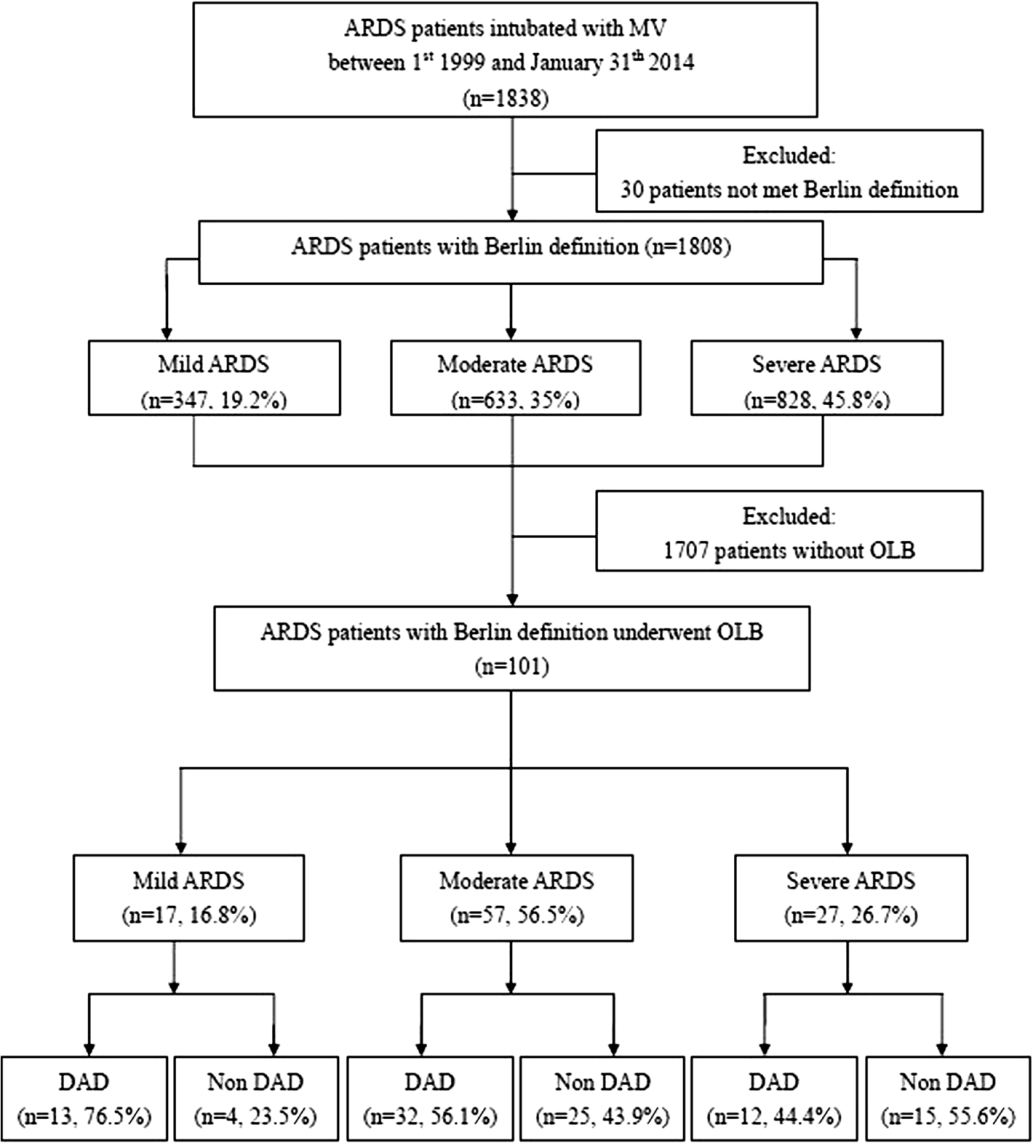
Flow chart of study patients. *ARDS* Acute respiratory distress syndrome, *MV* mechanical ventilation, *OLB* open lung biopsy, *DAD* diffuse alveolar damage

**Fig. 2:**
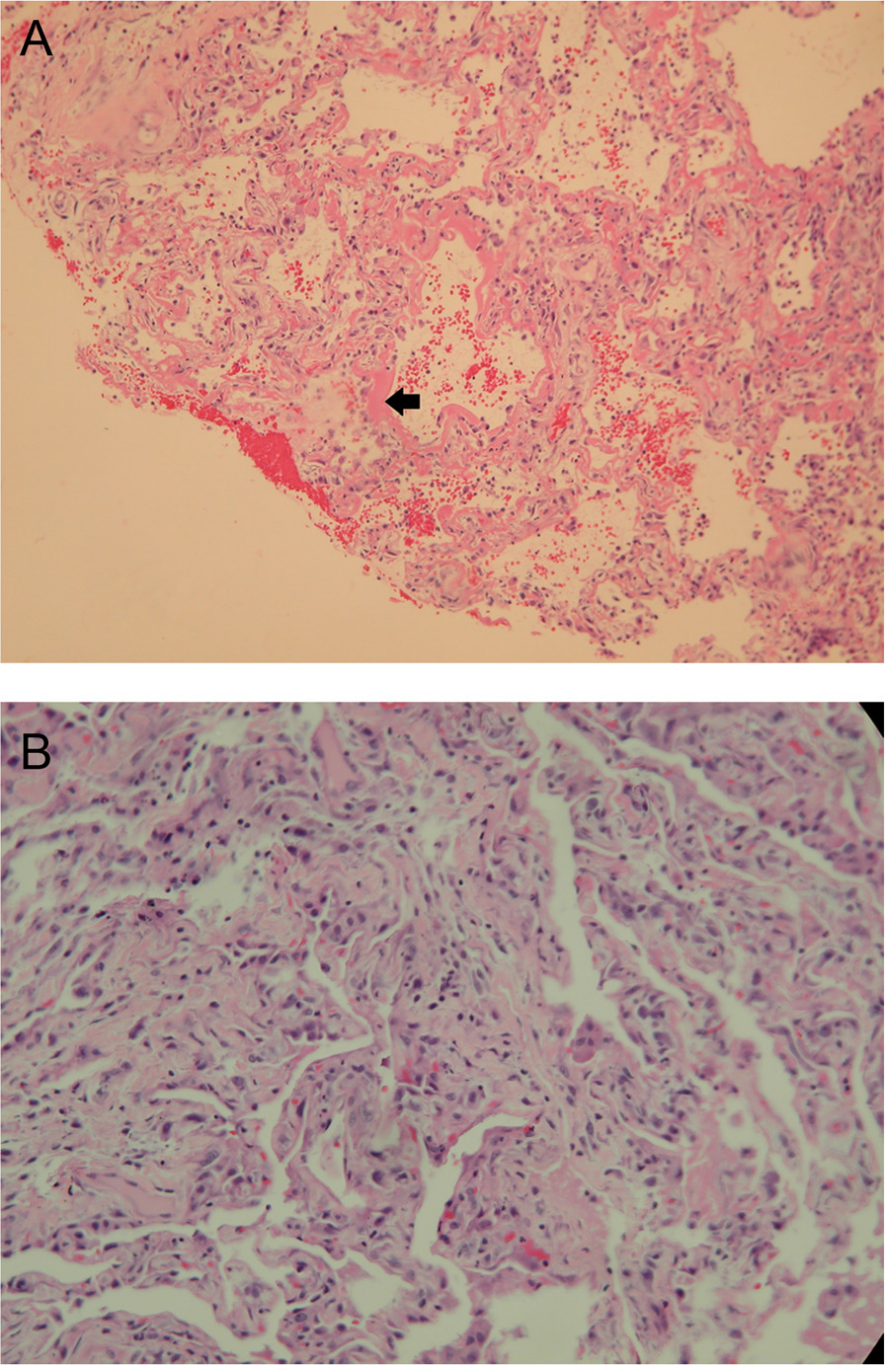
Pathological findings of diffuse alveolar damage. **a** Diffuse alveolar damage in the acute phase. The interstitium is edematous. Hyaline membrane (*arrow*) is seen lining the alveolar ducts (hematoxylin and eosin stain, ×100). **b** Diffuse alveolar damage in the organizing phase. The interstitium is thickened with organizing connective tissue. Prominent type 2 pneumocyte hyperplasia is seen (hematoxylin and eosin stain, ×200)

From 1 January 1999 to 31 January 2014, 1838 patients were admitted to our ICUs with a diagnosis of ARDS, of whom 101 had undergone open lung biopsy, and the overall hospital mortality rate was 60.4 % (Fig. 1). Of the 1838 screened patients, 30 patients were excluded because the symptom onset was more than 1 week ago and had not met Berlin criteria. Among the 101 patients who met the Berlin definition for ARDS, most patients were classified as having moderate ARDS (n = 57, 56.5 %), followed by severe ARDS (n = 27, 26.7 %), and mild ARDS (n = 17, 16.8 %). The proportions of DAD were 76.5 % (13/17) in mild, 56.1 % (32/57) in moderate and 44.4 % (12/27) in severe ARDS and there were no significant differences among these three groups (*p* = 0.113). According to the severity on the biopsy day, the proportions of DAD were 72 % (13/18) in mild, 56.9 % (33/58) in moderate and 44 % (11/25) in severe ARDS and there were no significant differences among these three groups (*p* = 0.113). Figure 2 shows the pathological findings of diffuse alveolar damage in the acute phase and organizing phase.

Baseline characteristics of these patients are shown in Table 1. There were more male patients with ARDS compared with female patients (64.4 % vs 35.5 %). Twenty-six patients were had immunocompromised status, including thirteen who had received chemotherapy within a month before the onset of ARDS, six who were recipients of HSCT, three who had HIV infection, three who had received long-term systemic corticosteroids, and one patient who had received a renal transplant. The average PaO_2_/FiO_2_ ratios on the day of ARDS diagnosis vs the day of biopsy were 142.4 ± 60.2 vs 148.7 ± 67.9, respectively.

**Table 1:** Baseline characteristics of patients with ARDS who had open lung biopsy (n = 101)

Characteristic	Value
Age	56.8 ± 16.8
Gender, number (male/female)	65/36
Immunocompromised, number	26 (26 %)
APACHE II score, ARDS diagnosis day	23.2 ± 5.5
SOFA score, ARDS diagnosis day	6.1 ± 3.2
PaO_2_/FiO_2_ (mm Hg), ARDS diagnosis day	142.4 ± 60.2
PEEP (cm H_2_O), ARDS diagnosis day	11.5 ± 2.1
SOFA score, biopsy day	7.0 ± 3.4
PaO_2_/FiO_2_ (mm Hg), biopsy day	148.7 ± 67.9
PEEP (cm H_2_O), biopsy day	11.8 ± 2.7
Tidal volume (mL), biopsy day	423.3 ± 99.6
Tidal volume (mL/predicted body weight)	8.3 ± 2.0
Duration from ARDS diagnosis to biopsy (days)	7.4 ± 7.1
Categories, ARDS diagnosis day, number (%)	101 (100 %)
Mild ARDS	17 (16.8 %)
Moderate ARDS	57 (56.5 %)
Severe ARDS	27 (26.7 %)
Categories, biopsy day, number (%)	101 (100 %)
Mild ARDS	18 (17.8 %)
Moderate ARDS	58 (57.4 %)
Severe ARDS	25 (24.8 %)

All values are expressed as number of patients (%) or mean ± SD. Abbreviations: *ARDS* Acute respiratory distress syndrome, *APACHE* Acute physiology and chronic health evaluation, *SOFA* Sequential organ failure assessment, *PaO*_*2*_ Arterial partial pressure of oxygen, *FiO*_*2*_ Fraction of inspired oxygen, *PEEP* Positive end-expiratory pressure

Sixty-nine patients (68.3 %) had an HRCT of the chest and 94 patients (93 %) underwent a flexible bronchoscopic examination to identify the etiology of ARDS before open lung biopsy. Among the 94 patients receiving bronchoscopic examination, eight patients did not undergo BAL due to severe hypoxemia during the procedure. Eighty patients (79.2 %) underwent open-lung biopsy in the operating room and 21 patients (21.8 %) received bedside open lung biopsy in the ICU. For open lung biopsy, VATS was used in 57 patients (56.4 %), and thoracotomy was performed in 44 patients (43.6 %). Thirty-eight patients (37.6 %) received corticosteroids at the time of open lung biopsy. Surgical complications were reported in 14 patients (13.9 %), but no complication resulted directly in death. Eight patients had persistent air leak after surgery, three patients had postoperative subcutaneous emphysema, two patients had transient hypotension, and one patient had a hemothorax.

All biopsy specimens exhibited abnormalities and provided sufficient evidence for pathological diagnosis. The pathological diagnoses of open lung biopsy are shown in Table 2. DAD was diagnosed in 57 patients (56.4 %); of these, there were 41 patients with DAD only and 16 patients with DAD associated with another condition. Of these 16 patients, 11 were classified as having infection (4 patients with pneumocystis jiroveci pneumonia, 3 with invasive fungal infection, 2 with *Staphyloccus aureus*, 1 with a viral infection and 1 with mycobacterium tuberculosis), 2 as having an interstitial lung pattern (both patients having organizing pneumonia) and 3 were classified as having miscellaneous infection (2 patients with metastatic adenocarcinoma and 1 with vasculitis). Forty-four patients did not have DAD and 10 of these patients were classified as having infection (1 patient with pneumocystis jiroveci pneumonia and cytomegalovirus pneumonia, 2 with pneumocystis jiroveci pneumonia, 2 with viral pneumonia, 2 with invasive fungal infection, 2 with bacterial pneumonia and 1 with mycobacterium tuberculosis), 18 patients had interstitial lung patterns (5 patients with usual interstitial pneumonia, 4 with organizing pneumonia, 2 with nonspecific interstitial pneumonia, 1 with desquamative interstitial pneumonia, 1 with hypersensitive pneumonitis and 5 with unclassified interstitial pneumonitis), and 16 patients were classified as having miscellaneous conditions (7 patients with fibrosis, 3 with leukemic infiltration, 2 with metastatic adenocarcinoma, 2 with vasculitis and 2 with lung edema). There were 21 pneumonia patients including 11 patients with DAD and 10 patients without DAD. The hospital mortality rate was significantly higher in patients with pneumonia and DAD (8/11) than in those with pneumonia without DAD (2/10) (72.7 % vs 20 %, *p* = 0.03).

**Table 2:** Pathological diagnosis of patients with ARDS who had open lung biopsy (n = 101)

Pathological diagnosis	Number (%)
DAD	57 (56 %)
DAD only	41
Infectious disease	11
Interstitial lung disease	2
Miscellaneous	3
Non-DAD	44 (44 %)
Infectious disease	11*
Interstitial lung disease	18
Miscellaneous	16

All values are expressed as number of patients (%) or mean ± SD. ^*^One pathology result included both pneumocystis jiroveci pneumonia and cytomegalovirus pneumonia. Abbreviations: *ARDS* Acute respiratory distress syndrome; *DAD* diffuse alveolar damage

According to the results of open lung biopsy, 49 patients had alterations to their therapy (48.5 %). These alterations included 16 patients who had steroid therapy introduced, 7 patients whose antimicrobial agent was changed, 1 patient who had chemotherapy introduced, and 25 patients in whom certain medications were discontinued, including withdrawal of antibiotics in viral pneumonia and metastatic carcinoma, and withdrawal of corticosteroids in bacterial pneumonia.

The hospital mortality rates were 64.7 % in mild ARDS, 61.4 % in moderate ARDS, and 55.6 % in severe ARDS and there was no significant difference between the three groups (*p* = 0.81). Table 3 shows the demographic and clinical characteristics of survivors vs non-survivors among our patients with ARDS. The SOFA score and PaO_2_/FiO_2_ ratio on the day of open lung biopsy were significantly different between survivors and non-survivors (SOFA score 5.2 ± 2.7 vs 8.1 ± 3.4, *p* <0.001; PaO_2_/FiO_2_ 167.6 ± 69.3 vs 136.3 ± 64.6, *p* = 0.023, respectively). The finding of DAD on pathological examination was significantly higher in non-survivors than survivors (67.2 % vs 40 %, *p* = 0.007).

**Table 3:** Demographic and clinical characteristics of survivors versus. non-survivors in patients with ARDS who had open lung biopsy

Characteristics	Survivors (n=40)	Non-survivors (n=61)	*P* value
Age	54.5 ± 15.4	58.3 ± 17.6	0.274
Gender (male/female)	23/17	42/19	0.244
Immunocompromised (%)	32.5	21.3	0.208
APACHE II score, diagnosis day	23.4 ± 5.3	23.1 ± 5.7	0.838
SOFA score, diagnosis day	5.4 ± 2.6	6.6 ± 3.5	0.06
PaO_2_/FiO_2_ (mm Hg), diagnosis day	136.5 ± 55.3	146.3 ± 63.4	0.427
SOFA score, biopsy day	5.2 ± 2.7	8.1 ± 3.4	<0.001*
PaO_2_/FiO_2_ (mm Hg), biopsy day	167.6 ± 69.3	136.3 ± 64.6	0.023*
PEEP (cm H_2_O), biopsy day	11.2 ± 2.7	12.2 ± 2.7	0.071
Tidal volume (mL), biopsy day	437.1 ± 86.0	413.4 ± 107.2	0.372
Duration from diagnosis to biopsy (days)	7.1 ± 6.4	8.1 ± 7.1	0.232
Operative complication rate (%)	5	18	0.056
DAD on biopsy (%)	40	67.2	0.007*
Treatment changes after biopsy (%)	60	41	0.061

All values are expressed as number of patients (%) or mean ± SD; *p values <0.05. Abbreviations: *ARDS* Acute respiratory distress syndrome, *APACHE* Acute physiology and chronic health evaluation; *SOFA* Sequential organ failure assessment, *PaO*_*2*_ Arterial partial pressure of oxygen, *FiO*_*2*_ Fraction of inspired oxygen, *PEEP* Positive end-expiratory pressure, *DAD* Diffuse alveolar damage

Univariate analysis was used to identify variables that have prognostic value for hospital mortality, and multivariate logistic regression analysis was used to identify variables that did not have significant prognostic value (Table 4). Identification of DAD on pathological examination (odds ratio 3.554, 95 % CI 1.385, 9.120; *p* = 0.008) and SOFA score on the biopsy day (odds ratio 1.424, 95 % CI 1.187, 1.707; *p* <0.001) were significantly and independently associated with hospital mortality. Regression coefficients for these variables were used to calculate a natural logarithm of the odds (logit) of the probability of death (p) as follows:

$$ \mathrm{logit}\ \left(\mathrm{p}\right) = -2.522 + \left(1.268 \times \mathrm{DAD}\right) + \left(0.353 \times \mathrm{SOFA}\ \mathrm{score}\right). $$

**Table 4:** Univariate and multivariate logistic regression analysis of clinical variables associated with hospital mortality

Parameter	Beta coefficient	Standard error	Odds ratio (95 % CI)	*p* value
Univariate logistic regression				
Age	0.013	0.012	1.014 (0.989, 1.038)	0.272
Male gender	−0.491	0.423	0.612 (0.267, 1.402)	0.246
Immunocompromised	0.575	0.460	1.778 (0.721, 4.381)	0.211
SOFA score, diagnosis day	0.130	0.071	1.139 (0.991, 1.307)	0.066
PaO_2_/FiO_2_, diagnosis day	0.003	0.003	1.003 (0.996, 1.010)	0.423
SOFA score, biopsy day	0.326	0.085	1.385 (1.173, 1.635)	<0.001*
PaO_2_/FiO_2_, biopsy day	−0.007	0.003	0.993 (0.987, 0.999)	0.03*
Treatment changes after biopsy	−0.770	0.415	0.463 (0.205, 1.044)	0.063
DAD on biopsy	1.123	0.423	3.075 (1.343, 7.039)	0.008*
Multivariate logistic regression				
DAD on biopsy	1.268	0.481	3.554 (1.385, 9.120)	0.008*
SOFA score, biopsy day	0.353	0.093	1.424 (1.187, 1.707)	<0.001*
Constant	−2.522	0.698	0.080	<0.001*

*p <0.05. In multivariate logistic regression, the Hosmer-Lemeshow test results (*χ*^2^ = 3.371, degrees of freedom = 8, *p* = 0.909). Abbreviations: *SOFA* Sequential organ failure assessment, *PaO*_*2*_ Arterial partial pressure of oxygen, *FiO*_*2*_ fraction of inspired oxygen, *DAD* diffuse alveolar damage

Demographic and clinical characteristics of patients with ARDS with DAD vs those without DAD are shown in Table 5. There were no clinical characteristics that could discriminate patients with DAD from those without DAD before open lung biopsy. Patients with ARDS and a pathologic diagnosis of DAD had a higher mortality rate than those without DAD (71.9 % vs 45.5 %, *p* = 0.007).

**Table 5:** Demographic and clinical characteristics of patients with ARDS with DAD versus patients without DAD

Characteristics	DAD (n = 57)	No DAD (n = 44)	*p* value
Age	56.6 ± 17.5	57.1 ± 16.1	0.876
Gender (male/female)	36/21	29/15	0.775
Immunocompromised (%)	28.1	22.7	0.543
APACHE II score, diagnosis day	22.6 ± 6.1	23.7 ± 5.1	0.506
SOFA score, diagnosis day	6.3 ± 3.7	5.9 ± 2.5	0.492
PaO_2_/FiO_2_ (mm Hg), diagnosis day	148.6 ± 62.0	134.1 ± 57.3	0.234
SOFA score, biopsy day	7.3 ± 3.9	6.6 ± 2.7	0.326
PaO_2_/FiO_2_ (mm Hg), biopsy day	158.5 ± 72.5	136.0 ± 59.9	0.099
PEEP (cm H_2_O), biopsy day	12.0 ± 3.0	11.5 ± 2.3	0.423
Tidal volume (mL), biopsy day	422.3 ± 95.4	422.4 ± 108.5	0.995
Duration from diagnosis to biopsy (days)	7.6 ± 6.7	7.7 ± 7.1	0.724
Operative complication rate (%)	10.5	15.9	0.423
Treatment changes after biopsy (%)	43.9	54.5	0.287
Hospital mortality (%)	71.9	45.5	0.007*

All values are expressed as number of patients (%) or mean ± SD; *p values <0.05. Abbreviations: *DAD* Diffuse alveolar damage, *ARDS* Acute respiratory distress syndrome, *APACHE* Acute physiology and chronic health evaluation; *SOFA* Sequential organ failure assessment, *PaO*_*2*_ Arterial partial pressure of oxygen, *FiO*_*2*_ Fraction of inspired oxygen, *PEEP* Positive end-expiratory pressure

## Discussion

In our study, DAD was found in 56.4 % of patients who had ARDS diagnosed using the Berlin definition and who underwent open lung biopsy. The overall hospital mortality rate was 60.4 %. DAD and SOFA scores obtained by pathological examination on biopsy day were significantly associated with hospital mortality. Demographic and clinical characteristics were not significantly different between patients with and without DAD patients before open lung biopsy.

The typical pathological finding of ARDS is DAD but the correlation between clinical criteria for ARDS and DAD is not well-understood [1, 9, 20, 21]. In an autopsy series, of the 127 patients who met the AECC criteria for ARDS, 66 % (84/127) were found to have DAD identified on pathological examination [21]. In another study based on autopsy data, DAD was documented in 43 % (35/82) of patients who met the AECC criteria for ARDS [31]. Another autopsy study by Thille et al., identified DAD on pathological examination in 45 % (159/356) of patients who met the clinical criteria for ARDS according to the Berlin definition at the time of death [23]. Furthermore, DAD was more frequently identified (69 %) in those who had severe ARDS for 72 hours or longer. In our open lung biopsy data, DAD was noted in 56.4 % (57/101) of all patients with ARDS and was more frequently found in patients with mild ARDS (76.5 %). This difference in frequency of DAD in patients with ARDS based on autopsy vs open lung biopsy may be explained by the different distribution of ARDS among the patient groups. The majority of patients had severe ARDS in the Thille group based on autopsy but had moderate ARDS in the present study based on open lung biopsy.

The percentage of patients with ARDS who were found to have DAD on open lung biopsy varies widely among studies. Papazian et al. reported results of a retrospective study of patients with ARDS who met AECC criteria and who underwent open lung biopsy [14]. They found that 13.8 % (5/36) of patients had DAD. In another study, Papazian and coworkers prospectively studied 100 patients with ARDS who fulfilled AECC criteria and who underwent open lung biopsy; 13 % were identified to have DAD on pathological examination [17]. In Patel’s study of open lung biopsy in patients with ARDS, 40 % (23/57) of patients had DAD on pathologic examination [15]. In our previous study, DAD was found in 29.3 % (12/41) of patients with early-stage ARDS, who were receiving open lung biopsy [16]. A recent study in non-resolving ARDS showed the DAD was found in 57.8 % (48/83) patients who had open lung biopsy [32]. In the present study of open lung biopsy, DAD was noted in 56.4 % (57/101) of patients with ARDS diagnosed using the Berlin definition. The wide variation in percentages of DAD in patients with ARDS (from 13.5 % to 57.84 %) based on open lung biopsy may be attributable to the different indications, definition and timing for open lung biopsy in each of the studies.

In this study, we demonstrated that a DAD identified on pathological examination correlated with increased mortality in patients with ARDS identified using the Berlin definition. In a study by Parambil et al., the hospital mortality rate in patients with DAD was 58 % in patients with ARDS compared to 17 % in patients without ARDS [31]. In a study of patients with ARDS (Papazian et al.), the factors predicting survival included female gender, organ system failure score on the day of biopsy, and biopsy result leading to the addition of a new drug [17]. Another open lung biopsy study was performed in mechanically ventilated patients with undiagnosed diffuse pulmonary infiltrates to determine factors independently associated with survival [33]. They found that the Charlson age-comorbidity index score, number of organ dysfunctions, and the PaO_2_/FiO_2_ ratio on the day of biopsy were associated with survival. A study in patients with non-resolving ARDS, who had open lung biopsy showed that the ICU mortality rate was higher in patients with DAD than in those without DAD (67 % vs 57 %), but this was not significantly different [32]. The present study demonstrated that patients with DAD had significantly increased hospital mortality compared to those without DAD (71.9 % vs 45.5 %, *p* = 0.007). The poor survival outcome in patients with DAD may imply more severe lung injury in patients with ARDS with vs without DAD. However, no clinical characteristic differentiated between patients with and without DAD before open lung biopsy (Table 5). Our study highlights the heterogeneity of patients with ARDS and discrepancy between the clinical and pathological definitions of ARDS. A distinct phenotype of patients with ARDS characterized by DAD needs to be further addressed due to it having worse outcomes.

A meta-analysis of open lung biopsy in patients with ARDS summarized that the surgical complication rate was 22 %; the most common complication was prolonged air leak, but mortality resulting from surgery was infrequent [34]. Meanwhile, open lung biopsy could offer a specific diagnosis in 84 % of patients and altered management in 73 % of patients. Our study revealed that the surgical complication rate was 13.9 % and management alteration rate was 48.5 % in these selected patients with ARDS. The survival advantage of open lung biopsy in ARDS is still not approved due to lack of a randomized controlled trial. Practically, it is important to assess the balance between the potential risk of surgical complications and diagnosis or treatment benefit in deciding to perform open lung biopsy.

There were several limitations of our study. First, it is a retrospective study in one referred medical center, which may limit the generalization to all other ICUs. Given the patients included retrospectively from the charts, some patients with ARDS may have been missed. In addition, the ICD-9 diagnosis code 518.82 excludes ARDS associated with trauma and surgery and includes all type of hypoxemic acute respiratory failure. Some patients with trauma-associated or surgery-associated ARDS may be missed in our study. However, it is one of the largest studies in patients with ARDS having open lung biopsy. Second, the decision to perform open lung biopsy was highly selective and only 5.5 % of patients with ARDS were referred for biopsy in this study. The results, therefore, are unlikely to be representative of all patients with ARDS. However, the potential for selection bias for patients with ARDS may be lower with open lung biopsy than with autopsy as the patients are alive. Third, only 85 % of patients had BAL before open lung biopsy. Some specific diagnoses may have been missed because their recognition depended on the availability of laboratory facilities. Fourth, the lung specimens were assessed by one pulmonary pathologist only and so the possibility of individual interpretation bias should be considered. Finally, there was no standard treatment for patients with some specific diagnoses, such as interstitial lung disease, after the biopsy result became available. Specific treatments such as corticosteroid therapy might have influenced the survival outcome in these patients.

## Conclusions

This retrospective study demonstrated that 56.4 % of selected patients with ARDS based on the Berlin definition and who underwent open lung biopsy were identified as having DAD. As a result of the moderate agreement between clinical and pathological diagnosis of ARDS, open lung biopsy may be considered in some patients to exclude or to clarify the diagnosis. According to the results of pathological examination, patients with DAD had poorer outcomes than patients without DAD. However, there was no clinical characteristic that discriminated between patients with and without DAD before open lung biopsy. In future studies of ARDS, the clinical therapeutic trial may focus on this subgroup owing to the high mortality among these patients. In addition to open lung biopsy, some non-invasive modalities such as serum biomarkers and HRCT may be used to identify this distinct type of patient with ARDS.

## Key messages

- This retrospective study demonstrated that 56.4 % of selected patients with ARDS based on Berlin definition, and who underwent open lung biopsy were found to have DAD.
- Patients with DAD identified on pathological examination had significantly higher hospital mortality than patients without DAD, and there was no clinical characteristic that differentiated between patients with and without DAD before open lung biopsy.
- In future studies, the clinical therapeutic trial may focus on patients with ARDS and DAD and investigate some non-invasive modalities to identify this subgroup of patients with ARDS.

## Abbreviations

AECC: American-European Consensus Conference
ALI: Acute lung injury
APACHE: Acute physiology and chronic health evaluation
ARDS: Acute respiratory distress syndrome
BAL: Bronchoalveolar lavage
CPAP: Continuous positive airway pressure
CXR: Chest x-ray
DAD: Diffuse alveolar damage
FiO_2_: Inspired oxygen fraction
HRCT: High-resolution computed tomography
HSCT: Hematopoietic stem cell transplantation
LIS: Lung injury score PaO_2_, Arterial partial pressure of oxygen
PEEP: Positive end-expiratory pressure
SOFA: Sequential organ failure assessment
VATS: Video-assisted thoracoscopic surgery
